# Painless Vaccination*This letter was written to Dr. Hutchins. As it concerns a method of painless vaccination lately described by him in this Journal, it is in place to publish it here.—Eds.

**Published:** 1898-03

**Authors:** J. H. Moore

**Affiliations:** Fair Play, S. C.


### PAINLESS VACCINATION.*

FAIR PLAY, S. C., January 28, 1898.

. . . I will give you my experience with your "new method of vaccinating," as originated by you and published in the November issue of THE JOURNAL. When I first read the report the plan seemed so feasible that I determined to try it. As an experiment I first tried it on myself and little girl, using only one side of the point on her arm and the other on my own, and I got beau-

---

*This letter was written to Dr. Hutchins. As it concerns a method of painless vaccination lately described by him in this JOURNAL, it is in place to publish it here.—EDS.

tiful results; and after that, when I could get two or more to be vaccinated at the same time, I would only use one side of the point to each arm, and out of thirty-six cases I have only had failures in two of them, and I took special pains to look after them in order to find out and test its efficacy. I have seen the other method employed, with about fifty per cent. of failures, so I am bound to think that yours is the best method yet used, for several reasons; as by the use of the liq. pot. you only take off enough of the epidermis, and do not have any bleeding to interfere with its taking, as you are liable to if you should go the least bit too deep with an instrument, and then it is practically painless, which makes it preferable for children, as they naturally have a horror of "cutting."

My little girl, three and a half years old, enjoyed the proceedings immensely. After dissolving the epidermis as you suggest, I rub it off with a rubber, and just before applying the virus I wash the denuded surface off with a wet rag or absorbent sponge, to be sure I get all the liq. pot. off, and then apply the virus, and with the point of the ivory I gently scratch over the surface and leave it to dry. Indeed, the whole proceeding is so simple it seems strange it was never thought of before. For my part I thank you for the method which I believe will be the universal one, if it is ever tried. It will be with me. Yours fraternally,

J. H. MOORE, M.D.

